# Association Between Thromboelastometry Identified Hypercoagulability and Thromboembolic Complications After Arthroplasty: A Prospective Observational Study in Patients With Obesity

**DOI:** 10.1177/10760296231199737

**Published:** 2023-10-09

**Authors:** Usha Gurunathan, Lily Chiang, Joel Hines, Bronwyn Pearse, Scott McKenzie, Karen Hay, Daniel Mullany, Harshal Nandurkar, Victoria Eley

**Affiliations:** 1 Department of Anaesthesia and Perfusion Services, 67567 The Prince Charles Hospital, Brisbane, QLD, Australia; 2 Faculty of Medicine, The University of Queensland, Brisbane, QLD, Australia; 3 Adult Intensive Care Unit, 67567 The Prince Charles Hospital, Brisbane, QLD, Australia; 4 Blood Management Unit, 67567 The Prince Charles Hospital, Brisbane, QLD, Australia; 5 Department of Cardiology, 67567 The Prince Charles Hospital, Brisbane, QLD, Australia; 6 56362 QIMR Berghofer Medical Research Institute, Brisbane, QLD, Australia; 7 Department of Haematology, 5392 Alfred Health, Melbourne, VIC, Australia; 8 Australian Centre for Blood Diseases, Monash University, Melbourne, VIC, Australia; 9 Department of Anaesthesia and Perioperative Medicine, 3883 The Royal Brisbane and Women's Hospital, Brisbane, QLD, Australia

**Keywords:** arthroplasty, hypercoagulability, obesity, perioperative, thromboelastometry, thromboembolism

## Abstract

The prothrombotic state of obesity can increase the risk of thromboembolism. We aimed to investigate if there was an association between baseline hypercoagulable rotational thromboelastometry (ROTEM) profile and thromboembolic complications in arthroplasty patients with obesity. Patients with a body mass index ≥ 25 kg/m^2^ and/or waist circumference ≥94 cm (M) and 80 cm (F) undergoing hip and knee arthroplasty had pre- and postoperative ROTEM. ROTEM values were compared by outcome status using an independent sample equal-variance *t*-test. Of the 303 total participants, hypercoagulability defined as extrinsically activated thromboelastometry maximum clot firmness *G* score ≥ 11 K dyne/cm^2^, was observed in 90 (30%) of the 300 participants with preoperative ROTEM assays. Clinically significant thromboembolic complications occurred in 5 (1.7%) study participants before discharge and in 10 (3.3%) by 90 days. These included 6 with pulmonary emboli, 3 with deep venous thrombus, and 1 with myocardial infarction. We found no evidence for an association between baseline hypercoagulability and incident thromboembolic events, analysis limited by the number of events. Postoperative decrease in platelets and an increase in fibrinogen were observed. ROTEM parameter changes differed across obesity categories.

## Introduction

Thromboembolic (TE) complications are infrequent, but a major cause of morbidity and mortality following lower limb total joint arthroplasty.^1^ The prevalence of obesity and morbid obesity has significantly increased in arthroplasty patients in the last decade. It is estimated that by 2029 at least 69% of total knee arthroplasty (TKA) and 55% of total hip arthroplasty (THA) patients in the United States will be obese or morbidly obese.^2,3^ Obesity is one of the risk factors for postarthroplasty venous thromboembolism (VTE).^4^ While body mass index (BMI) is used to define obesity, metabolic syndrome identifies individuals at a higher risk for VTE^5^ and is described using waist circumference as the obesity metric.^6^

Obesity increases prothrombotic tendency due to chronic low-grade inflammation and impaired fibrinolytic activity.^7^ Central obesity accelerates atherosclerotic vascular damage and increases the susceptibility to adverse cardiovascular events.^8,9^ General adiposity can also increase the risk of myocardial infarction irrespective of the presence of metabolic syndrome.^10^ An association between VTE and arterial thrombotic risk was demonstrated by the Nateglinide and Valsartan in Impaired Glucose Tolerance Outcomes Research (NAVIGATOR) study that evaluated 9306 patients with impaired glucose tolerance.^11^ In that study, those with VTE were older, obese, and had higher rates of death, myocardial infarction, and stroke in the long term compared to those without VTE (10.7% vs 5.9%; *P* < .001; adjusted hazard ratio 2.12; 95% confidence interval, 1.36-3.31; *P* < .001).^11^

Patients with obesity have been shown to exhibit hypercoagulability compared to normal-weight patients in a trauma setting.^12^ Hypercoagulability has been demonstrated to be the strongest independent predictor for VTE following arthroplasty in a large database study of 1.7 million patients.^13^ Identifying hypercoagulability in obesity,^14,15^ and predict patients at higher risk of developing postoperative TE complications^14,16^ have been possible with point-of-care viscoelastic assays such as rotational thromboelastometry (ROTEM). Individualized TE risk assessment may reduce the complications associated with inappropriate thromboprophylaxis following arthroplasty.^13^ However, this application has not been well-researched in obese arthroplasty patients. Hence, we aimed to investigate the differences in baseline hypercoagulability identified by ROTEM between those with and without TE complications following elective lower limb arthroplasty in patients with obesity, defined by their BMI or their waist circumference. Our hypothesis was that hypercoagulability observed in obesity will be associated with increased TE risk following arthroplasty.

## Methods

### Design

This prospective observational study was conducted between October 2019 and August 2021 at a tertiary hospital where ∼ 800 primary THA and TKA are performed annually. Hospital ethics committee approval and individual written informed consent were obtained. The study was prospectively registered with the Australian New Zealand Clinical Trials Registry (No: ACTRN12615000825550).

### Inclusion and Exclusion Criteria

Patients were included in the study if they underwent elective primary THA and TKA and had a BMI ≥ 25 kg/m^2^ and/or waist circumference ≥ 94 cm (for males) and 80 cm (for females).^6,17^ Patients with a history of thrombophilia, other reasons for hypercoagulability (active malignancy, prolonged immobility, oral contraceptive pills, hormone replacement therapy, history of VTE in the last 12 months, and nephrotic syndrome) and on long-term therapeutic anticoagulation for medical indications were excluded.

### Data Collection and Management Protocol

Participant characteristics, clinical details, and laboratory results (coagulation tests, preoperative high-sensitive C-reactive protein [hs-CRP] levels, and lipid profile) were collected preoperatively. The current use of antiplatelet and anticoagulants was documented. Anthropometric indices (BMI, waist, neck, and hip circumferences, and waist-to-hip ratio) were measured on the day of the procedure applying standardized techniques.^18,19^ The participants were categorized according to the World Health Organisation (WHO) criteria.^20^ Those with normal BMI were included if they met the waist circumference criteria: ≥ 94 cm (for males) and 80 cm (for females).^18^ Metabolic syndrome was defined as per the 2009 international consensus statement.^6^

On the day of surgery, baseline ROTEM and troponin measurements were performed prior to anesthetic induction. Collected samples were immediately transferred by trained personnel to 1 ×  citrated vacutainer (2.7 mL containing 3.2% sodium citrate) and processed within 15 min of collection. The ROTEM *sigma* analyzer™ (Tem Innovations GmbH, Munich, Germany) is a fully automated cartridge thromboelastometry that provides information on clot kinetics. Clotting time (CT), clot formation time (CFT), amplitude at 10 min (A10), maximum clot firmness (MCF) in InTEM (intrinsically activated thromboelastometry), ExTEM (extrinsically activated thromboelastometry), and FibTEM (fibrin-based thromboelastometry) assays were measured. Concentrations of cardiac troponin I were measured from serum samples using the Beckman Coulter High-Sensitive Troponin I (hs-cTnI) assay.

Intraoperative management of the patients was left to the discretion of the treating team. Standard thromboprophylaxis practices included intermittent sequential compression devices until discharge and the provision of graduated compression stockings for 6 weeks. Pharmacological thromboprophylaxis was prescribed as per our hospital guidelines and documented for each participant. Details of any transfusion, postoperative disposition, and length of hospital stay were collected for the study. The participants had daily follow-ups until discharge and telephone follow-ups up until 30-day and 90-day time points. Postoperative blood tests included ROTEM assays on the postoperative day (POD) 2 or 3, conventional coagulation assays and platelet count (POD 1), hs-cTnI (POD 1, 2, and 3 on the first 125 participants), and D-dimer (POD 3). These were collected and processed within 2 h of collection. The treating team and outcome assessors were blinded to the ROTEM, troponin, and D-dimer test results until the 90-day follow-up. Diagnosis and management of complications were left to the discretion of the treating team.

### Endpoints

The primary endpoint was in-hospital TE complications defined as a composite of myocardial infarction (MI), symptomatic deep venous thrombosis (DVT) and pulmonary embolism (PE), ischaemic stroke, nonfatal cardiac arrest, and cardiovascular death (defined as death due to MI, stroke, PE, heart failure, or cardiac arrhythmia). Secondary endpoints were perioperative myocardial injury, all-cause mortality, and thrombotic complications listed above at 30 days and 3 months. Myocardial injury was diagnosed based on hs-cTnI levels with sex-specific 99th percentile cut-offs of ≥10 ng/L (female) and ≥20 ng/L (male) with an increase from baseline levels of ≥5 units (for hs-cTnI) without associated ischaemic signs or symptoms.^21^ Confirmatory tests included: for myocardial infarction: ECG changes and biomarkers; for DVT: D-dimers and duplex ultrasonography; for PE: ventilation-perfusion scanning/computed tomography pulmonary angiography (CTPA); and for ischaemic stroke: cerebral imaging (noncontrast CT or CT angiography).

Hypercoagulability was determined using the ExTEM MCF and MCF *G* score. *G* score (shear modulus strength) was derived with the formula: [(5000 × MCF)/(100 − MCF)]/1000 and expressed as K dyne/cm^2^.^22,23^ A hypercoagulable profile was primarily defined as an ExTEM *G* score ≥ 11 K dyne/cm^2^,^15,22^ (definition 1). An alternative definition used in other studies^24,25^ was also explored. This was characterized by shorter CT or CFT (acceleration of clot propagation) and/or higher MCF (increased clot strength) than the control values in the assays, that is one or more of ExTEM CT < 50 s, ExTEM CFT < 46 s, InTEM CT < 161 s, InTEM CFT < 62 s, ExTEM MCF > 72 mm, InTEM MCF > 69 mm, FiBTEM MCF > 21 mm^26^ (definition 2).

### Sample Size Calculation

We estimated that the incidence of postoperative TE complications expected in overweight/obese surgical patients would be 5% based on the 4% complication rate reported in patients of normal BMI.^27^ A clinically meaningful difference in MCF values between those with and without TE complications was determined to be 3 units and the assumed standard deviation of MCF was 4 units.^25^ Based on these assumptions we calculated a sample size of 300 participants comprising sample sizes of 285 (without TE complications) and 15 (with TE complications) that would achieve 80% power to reject the null hypothesis of equal means using a two-sample equal-variance *t*-test at a 5% significance level.

### Statistical Analyses

Outcomes of interest were described as proportions with Agresti-Coull 95% confidence intervals. Mean MCF and MCF G-scores were compared by outcome status using an independent sample equal-variance *t*-test. Differences in patient characteristics by baseline hypercoagulability (*G* score ≥ 11 K dyne/cm^2^) and WHO BMI classification were explored posthoc. Variables were summarized as mean (SD) (for continuous) or frequency (%) (for categorical) and compared between BMI groups using ANOVA or Kruskal-Wallis test (continuous) or Pearson's chi-square or Fisher's exact test (categorical) as appropriate. Analyses were performed using the Stata statistical software package (Version 15).

## Results

**Figure 1.**
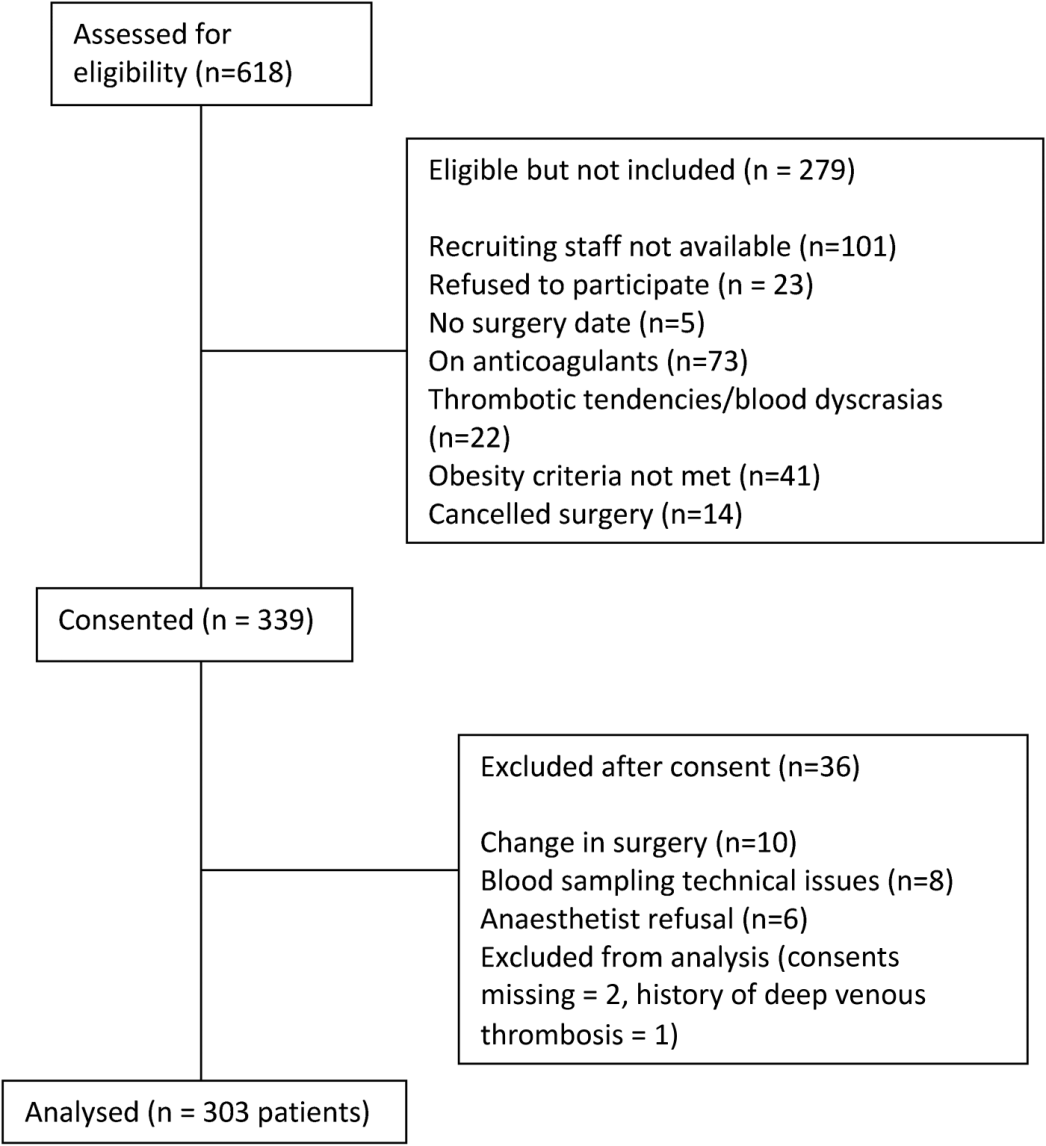
Consolidated standards of reporting trials (CONSORT) flow diagram of participant selection in the study.

A total of 303 patients, 171 females and 132 males, with a mean age of 66.6 (SD 9.5) years undergoing primary elective THA or TKA were included. Figure 1 demonstrates the reasons for participant exclusion. Median (IQR) BMI of the participants was 33.7 (30.3-37.2) kg/m^2^; mean (SD) waist circumference was 107.7 (12.1) cm and waist to hip ratio (WHR) was 0.9 (0.1). There were 68 (22.4%) participants with a BMI between 25 and 29.9 kg/m^2^ and 232 (76.7%) with a BMI ≥ 30 kg/m^2^. Of the 303 participants, 204 (67.3%) underwent TKA compared to 99 (32.7%) who underwent THA. The median (IQR) length of hospital stay following surgery was 4 (3-4) days. Forty-nine (16%) patients were taking preoperative aspirin. Regarding in-hospital thromboprophylaxis, the majority received aspirin or enoxaparin, and 17(5.6%) were changed from either aspirin or enoxaparin to another antithrombotic agent as per their clinician's usual practice or due to the development of in-hospital complications (for instance, rivaroxaban due to PE).

Our primary outcome, in-hospital TE complications occurred in 5 (1.7%) participants (3 in THA and 2 in TKA patients). Four female patients had symptomatic PE (POD 2, 3, 4, and 7) and one male patient developed swelling of the left knee (POD 3). Ultrasound finding in the male patient was suggestive of a nonocclusive thrombus of the mid-segment of a posterior tibial vein due to lack of compressibility. This patient was commenced on rivaroxaban and a repeat ultrasound in 3 weeks’ time was unremarkable. There were 21 participants (n = 125; 16.8%) with in-hospital myocardial injury. Those with TE complications were older, however, there was no evidence of differences in BMI (continuous or categorical) or ASA categories between those with and without TE complications (Table 1).

**Table 1.** Characteristics of the Participants by the Thromboembolic Complication Status at 3 Time Points, In-Hospital, 30 Days, and 90 Days Postsurgery.

Exposures of interest	In-hospital TE Complications (n = 303)		30-day TE Complications (n = 303)		90-day TE Complications (n = 303)		
	No (n = 298)	Yes (n = 5)	No (n = 294)	Yes (n = 9)	No (n = 293)	Yes (n = 10)	*P* value
Age (years)^a^	66.5 (9.5)	73.0 (10.5)	66.4 (9.5)	73.7 (7.9)	66.4 (9.5)	72.8 (7.9)	.037
Sex, female^c^	167 (56.0)	4 (80.0)	166 (56.5)	5 (55.6)	166 (56.7)	5 (50.0)	1.00
BMI (kg/m^2^)^b^	33.8 (30.4-37.3)	29.1 (27.5-31.9)	33.9 (30.4-37.3)	31.6 (28.9-32.5)	33.9 (30.4-37.3)	31.8 (28.9-35.5)	.17
Waist circumference (cm)^a^	108 (12.0)	97.1 (13.5)	108 (12.0)	102 (12.0)	108 (12.1)	103 (11.9)	.25
Hip circumference (cm)^b^	113.0 (106.0-123.0)	103 (101.0-133.0)	113.0 (106.0-123.0)	104.0 (101.0-128.0)	113.0 (106.0-123.0)	108 (101.0-128.0)	.36
Neck circumference (cm)^a^	39.3 (3.9)	38.5 (3.2)	39.3 (3.9)	38.5 (2.7)	39.3 (3.9)	39.1 (3.1)	.87
WHR^a^	0.9 (0.1)	0.8 (0.1)	0.9 (0.1)	0.9 (0.2)	0.9 (0.1)	0.9 (0.2)	.27
Surgery^c^							.73
THA	96 (32.2)	3 (60.0)	95 (32.3)	4 (44.4)	95 (32.4)	4 (40.0)	
TKA	202 (67.8)	2 (40.0)	199 (67.7)	5 (55.6)	198 (67.6)	6 (60.0)	
ASA status > 2^c^	127 (42.6)	4 (80.0)	126 (42.9)	5 (55.6)	126 (43.0)	5 (50.0)	.49
Baseline CRP (mg/L)^b^	2.8 (1.5-6.5)	1.5 (1.5-2.0)	2.8 (1.5-6.5)	1.8 (1.5-3.0)	2.8 (1.5-6.5)	2.0 (1.5-3.3)	.34
D-dimer (μg/mL)^b^	1.6 (1.3-2.1)	1.9 (1.8-3.6)	1.6 (1.3-2.1)	1.9 (1.3-3.6)	1.6 (1.3-2.1)	1.8 (1.3-3.6)	.36
Baseline ExTEM *G* score (K dynes/cm^2^)^a^	9.84 (2.13)	8.45 (2.53)	9.86 (2.14)	8.50 (1.87)	9.86 (2.14)	8.54 (1.77)	.055
Baseline hypercoagulability^c,d^ (n = 300)							
Definition 1	89 (30.2)	1 (20.0)	89 (30.6)	1 (11.1)	89 (30.7)	1 (10.0)	.29
Definition 2	140(47.5)	3 (60.0)	139 (47.8)	4 (44.4)	139 (47.9)	4 (40.0)	.75
In-hospital thromboprophylaxis							
Aspirin	221 (74.2)	1 (20.0)	217 (73.8)	5 (55.6)	216 (73.7)	6 (60.0)	.002
Enoxaparin	61 (20.5)	0 (0.0)	61 (20.7)	0 (0.0)	61 (20.8)	0 (0.0)	
Rivaroxaban	2 (0.7)	1 (20.0)	2 (0.7)	1 (11.1)	2 (0.7)	1 (10.0)	
Combination	14 (4.7)	3 (60.0)	14 (4.8)	3 (33.3)	14 (4.8)	3 (30.0)	

Abbreviations: ASA, American Society of Anesthesiologists; BMI, body mass index; CRP, C reactive protein; TE, thromboembolic; THA, total hip arthroplasty; TKA, total knee arthroplasty; WHR, waist-to-hip ratio.

^a^ Values are in mean (SD) with *P* values from unpaired *t*-test.

^b^ Or median (IQR) with *P* values from Wilcoxon's rank-sum test

^c^ n (%) with *P* values from Fisher's exact test.

^d^ Definition:1; *G* score ≥ 11 K dyne/cm^2^; Definition 2: shortened CT or CFT and/or higher MCF compared to control values.^20^

An additional 5 patients (1 underwent THA and 4 underwent TKA) developed TE complications during the 90-day follow-up period. Of them, 4 developed VTE complications between discharge and 30 days of surgery (2 PEs and 2 DVTs). A 65-year-old male developed an anterior ST-elevation myocardial infarction (STEMI) requiring an urgent percutaneous coronary intervention (PCI) with a drug-eluting stent (DES) between 30 and 90 days of surgery. Overall, this resulted in a TE complications rate of 3% (95% CI: 1.5-5.6; n = 9) within 30 days and 3.3% (95% CI: 1.7-6.0; n = 10) within 90 days following surgery.

Preoperative ROTEM assays were performed on 300 (of the 303) participants. We did not observe any statistical evidence of differences in baseline ExTEM MCF, any other ROTEM parameter, and conventional coagulation parameters between those with and without in-hospital TE complications 30-day or 90-day TE complications (Table 1). Mean baseline MCF *G* score was 8.45 (SD: 2.5) K dyne/cm^2^ in those with in-hospital VTE compared to 9.8 (SD:2.1) K dyne/cm^2^ in those without (*P* = .15) (Table 1 and Supplemental Table 1).

Ninety (30%) participants demonstrated hypercoagulability (*G* score of 11 ≥ K dyne/cm^2^) (Table 2). These participants had higher median BMI (*P* = .013) and hip circumference (*P* = .005) and included more females (*P* < .001) compared to those with a *G* score < 11 K dyne/cm^2^. Baseline platelets, fibrinogen level, and CRP level were on average higher in hypercoagulable patients (*P* < .001 for all) (Table 2). Due to the overall low event rate, neither the association nor the predictive value of ROTEM hypercoagulability for TE complications could be calculated.

**Table 2.** Summary Statistics of the Participants by Baseline hypercoagulability e^e^ (n = 300).

Parameters	*G* Score < 11 K dyne/cm^2^ (n = 210)	*G* Score ≥ 11 K dyne/cm^2^ (n = 90)	*P* Value
Age (years)^a^	66.7 (9.5)	66.5 (9.6)	.86
Sex, female^c^	95 (45.2)	73 (81.1)	<.001
ASA status > 2^c^	83 (39.5)	46 (51.1)	.063
BMI (kg/m^2^)^b^	33.1 (29.5-36.4)	34.7 (31.7-38.5)	.013
Waist circumference (cm)^a^	108 (12.1)	107 (12.1)	.34
Hip circumference (cm)^b^	111 (106-120)	116 (109-127)	.005
Neck circumference (cm)^a^	39.6 (4.0)	38.6 (3.7)	.049
WHR^a^	1.0 (0.1)	0.9 (0.1)	<.001
Metabolic syndrome^c,e^	89 (42.4)	51 (56.7)	.023
Surgery^c^			.94
THA	69 (32.9)	30 (33.3)	
TKA	141 (67.1)	60 (66.7)	
Comorbidities			
Active cancer^c^	15 (7.1)	8 (8.9)	.60
Current smoking^d^	10 (4.8)	1 (1.1)	.18
CCF^d^	4 (1.9)	3 (3.3)	.43
IHD^c^	14 (6.7)	7 (7.8)	.73
COPD^c^	9 (4.3)	9 (10.0)	.056
OSA^c^	45 (21.4)	21 (23.3)	.72
CVA^d^	8 (3.8)	4 (4.4)	.76
CKD^c^	18 (8.9)	10 (11.2)	.53
HTN^c^	116 (55.2)	57 (63.3)	.19
DM^c^	40 (19.0)	20 (22.2)	.53
Baseline CRP (mg/L)^b^	2.4 (1.5-5.2)	4.6 (2.2-9.6)	<.001
Baseline platelets (× 10^9^/L)^b^	234 (195.0-266.0)	297 (246.0-327.0)	<.001
Baseline INR^b^	1.0 (0.9-1.0)	1.0 (0.9-1.0)	.53
Baseline PT (s)^b^	11.0 (11.0-12.0)	11.0 (11.0-12.0)	.47
Baseline APTT (s)^b^	29.0 (26.5-31.0)	29.5 (28.0-32.0)	.032
Baseline fibrinogen (g/L)^b^	3.2 (2.8-3.7)	3.8 (3.5-4.3)	<.001

Abbreviations: ASA, American Society of Anesthesiologists; CCF, congestive cardiac failure; CKD, chronic kidney disease; COPD, chronic obstructive pulmonary disease; CRP, C-reactive protein; DM, diabetes mellitus; HTN, hypertension; IHD, ischaemic heart disease; OSA, obstructive sleep apnoea; THA, total hip arthroplasty; TKA, total knee arthroplasty; ExTEM, extrinsically activated thromboelastometry; MCF, maximum clot firmness.

^a^ Values are in mean (SD) with *P* values from unpaired *t*-test.

^b^ Median (IQR) with *P* values from Wilcoxon's rank-sum test.

^c^ Or n (%) with *P* values from Pearson's chi-square test.

^d^ Or Fisher's exact test.

^e^ Defined as ExTEM MCF *G* score ≥ 11 K dyne/cm^2^.

^f^ International consensus definition.

There were perioperative (albeit nonsignificant) changes consistent with an increasing hypercoagulability, greater in those who developed in-hospital VTE compared to those who did not. However, patients with VTE, had lower mean (SD) POD 3 platelet counts (151 [38] vs to 211 [52] × 10^9^/L; *P* = .011) and experienced more marked percentage reduction from baseline (28% vs 16%; *P* = .022) compared in those without VTE (Supplemental Table 1). This trend in POD 3 platelet counts was also evident in patients who developed 30- and 90-day TE (*P* = .015 and *P* = .045, respectively).

A posthoc exploratory analysis of the differences across the obesity categories was undertaken. Participants with higher BMI were on average younger (*P* = .024) and included a higher percentage of females (*P* < .001), more undergoing TKA (*P* < .001), and more with diabetes (*P* = .041). Median CRP levels increased progressively with the increase in BMI (*P* = .002) (Supplemental Table 2). Regarding coagulation parameters, baseline median fibrinogen level showed a progressive increase with BMI category (*P* < .001). Compared to baseline levels, median fibrinogen levels were higher on POD 3, and this perioperative increase was also different between BMI categories (*P* = .002) (Table 3). Similarly, ExTEM, FibTEM, A10 MCF, and InTEM MCF demonstrated a trend of progressive increase in clot strength with increasing BMI. ExTEM MCF G score was positively correlated with BMI, both at baseline (*P* = .027) and POD3 (*P* = .003). The prevalence of baseline hypercoagulability increased with BMI when defined by *G* score criteria (*P* = .040) (Table 3).

**Table 3.** Comparison of Coagulation Parameters in the Study Participants Across BMI Categories.

Parameter	BMI				*P* Value
	<30 kg/m^2^ and/or High Waist Circumference^b^ (n = 71)	30 to 34.99 kg/m^2^ (n = 104)	35 to 39.99 kg/m^2^ (n = 93)	> 40 kg/m^2^ (n = 35)	
Baseline values					
Platelets × 10^9^/L	238.0 (210.0-286.0)	246.0 (214.0-295.0)	244.0 (201.0-289.0)	249.0 (227.0-299.0)	.48
Fibrinogen, g/L	3.0 (2.6-3.6)	3.4 (2.9-3.8)	3.7 (3.0-4.0)	3.7 (3.2-4.4)	<.001
ExTEM^e^					
CT (s)	65.0 (57.0-72.0)	64.0 (60.0-70.0)	64.0 (60.0-70.0)	65.0 (60.0-68.0)	.97
CFT (s)	76.0 (63.0-89.0)	70.0 (58.0-83.0)	66.0 (56.0-86.0)	63.0 (58.0-80.0)	.069
A10 (mm)	55.0 (51.0-58.0)	57.0 (53.0-61.0)	58.0 (52.0-62.0)	59.0 (55.0-60.0)	.041
MCF (mm)	64.0 (62.0-67.0)	67.0 (63.0-70.0)	67.0 (62.0-70.0)	68.0 (64.0-70.0)	.010
InTEM^e^					
CT (s)	171.0 (161.0-183.0)	176.0 (162.0-185.0)	174.0 (156.0-185.0)	177.0 (164.0-196.0)	.17
CFT (s)	76.0 (65.0-93.0)	73.0 (59.0-90.0)	69.0 (58.0-89.0)	68.0 (58.0-81.0)	.31
A10 (mm)	53.0 (50.0-57.0)	55.0 (50.0-58.0)	56.0 (49.0-59.0)	56.0 (52.0-59.0)	.22
MCF (mm)	61.0 (59.0-65.0)	63.0 (60.0-66.0)	64.0 (58.0-67.0)	64.0 (61.0-67.0)	.035
FibTEM^e^					
CT (s)	65.0 (59.0-70.0)	64.0 (60.0-68.0)	63.0 (59.0-68.0)	65.0 (62.0-68.0)	.55
CFT (s)^c^	1033.5 (604.0-1597.0)	657.0 (206.0-1559.0)	840.5 (412.0-1933.0)	383.0 (251.0-819.0)	.39
A10 (mm)	14.0 (12.0-16.0)	15.0 (13.0-17.0)	17.0 (14.0-19.0)	16.0 (14.0-19.0)	<.001
MCF (mm)	15.0 (13.0-17.0)	16.0 (14.0-19.0)	18.0 (15.0-21.0)	18.0 (15.0-21.0)	<.001
Baseline hypercoagulability, (n = 300)^a,d^					
Definition 1	12 (16.9)	35 (34.0)	30 (33.0)	13 (37.1)	.040
Definition 2	26 (36.6)	47 (45.6)	50 (54.9)	20 (57.1)	.078
Postoperative values					
Platelets × 10^9^/L	195.5 (163.0-246.0)	206.0 (173.5-242.0)	204.0 (176.0-243.0)	225.0 (190.0-248.0)	.21
Fibrinogen, g/L^c^	7.3 (6.1-8.3)	7.9 (6.9-9.1)	7.1 (6.5-8.0)	7.7 (6.3-9.1)	.004
ExTEM^c^					
CT (s)	65.0 (59.0-71.0)	64.0 (58.0-69.0)	65.0 (59.0-74.0)	64.0 (59.0-68.0)	.70
CFT (s)	49.0 (42.0-60.0)	45.5 (40.0-52.0)	47.0 (42.0-55.0)	45.0 (39.0-49.0)	.11
A10 (mm)	64.0 (60.0-66.0)	66.0 (63.0-69.0)	65.0 (61.5-68.0)	65.5 (64.0-67.0)	.005
MCF (mm)	70.0 (67.0-72.0)	72.0 (70.0-75.0)	72.0 (68.0-74.0)	72.5 (71.0-74.0)	.003
InTEM^c^					
CT (s)	162.0 (154.0-170.0)	161.5 (147.0-172.0)	163.0 (152.5-170.0)	159.0 (153.0-169.0)	.86
CFT (s)	51.0 (47.0-65.0)	47.0 (41.0-53.0)	49.0 (44.0-57.5)	47.0 (42.0-54.0)	.023
A10 (mm)	60.0 (56.0-63.0)	62.0 (60.0-66.0)	61.0 (58.0-65.0)	63.0 (61.0-65.0)	.006
MCF (mm)	67.0 (64.0-70.0)	70.0 (67.0-72.0)	69.0 (65.0-71.0)	70.0 (68.0-72.0)	<.001
FibTEM^c^					
CT (s)	65.0 (59.0-72.0)	64.0 (58.0-71.0)	64.0 (58.0-69.5)	61.5 (58.0-68.0)	.54
CFT (s)	111.0 (68.5-183)	72.0 (56.0-125.0)	96.0 (66.0-177.0)	90.0 (63.0-131.0)	.031
A10 (mm)	25.0 (22.0-28.0)	27.0 (25.0-30.0)	26.0 (23.5-28.5)	26.0 (24.0-28.0)	.011
MCF (mm)	27.0 (25.0-32.0)	31.0 (28.0-33.0)	29.0 (26.5-32.0)	28.0 (27.0-32.0)	.015
Percent increase in fibrinogen	135 (86-170)	128 (87-183)	95 (70-130)	100 (79-143)	.002

Abbreviations: A10, amplitude at 10 min; BMI, body mass index; CT, clotting time; CFT, clot formation time; MCF, maximum clot firmness; ExTEM, extrinsically activated thromboelastometry; InTEM, intrinsically activated thromboelastometry; FibTEM, fibrin-based thromboelastometry.

^a^ Values are median (IQR) with *P* values from Wilcoxon's rank-sum test or ^a^n (%) with *P*-values from Pearson's chi-squared test.

^b^ High waist circumference: >80 cm (female); >94 cm (male).

^c^ Missing data > 15%.

^d^ Definition: 1: *G* score ≥ 11 K dyne/cm^2^; Definition 2: shortened CT or CFT and/or higher MCF compared to control values.

^e^ ROTEM reference ranges ExTEM: CT: 50 to 80 s; CFT: 46 to 149 s; A10: 43 to 63 mm; MCF: 55 to 72 mm; InTEM: CT: 161 to 204 mm; CFT: 62 to 130 s; A10: 43 to 62 mm; MCF: 51 to 69 mm; FibTEM: CT: 46 to 84 s; CFT: not reported; A10: 6 to 21 mm; MCF: 6 to 21 mm.

## Discussion

In this study, thromboembolic complications occurred in 1.7% of the patients before discharge, in 3% within 30 days, and in 3.3% within 90 days of primary elective THA/TKA with routine thromboprophylaxis. This included 6 patients with PE, 3 with DVT, and 1 patient with MI. Baseline hypercoagulability (ExTEM *G* score criteria), was observed in 30% of the study participants. However, there was no evidence of any association between preoperative hypercoagulability and postoperative TE complications.

Viscoelastic assays are considered superior to conventional coagulation tests in detecting hypercoagulability and predicting thromboembolic events in surgical patients.^28^ A viscoelastic hypercoagulable state is associated with 3.6-fold increased odds of developing postoperative TE complications and these assays are reported to have a high specificity of 76% for these events.^28^ In our study, only one of the 90 who showed baseline hypercoagulability developed TE complications in the 90-day follow-up period. There may be several explanations for our observations. We included only symptomatic and confirmed TE complications as endpoints similar to previous studies including the CRISTAL trial,^16,29^ due to the limited real-world clinical relevance of routine screening following arthroplasty. In fact, even routine ultrasound screening can miss up to 50% of DVT following major orthopedic surgery.^30^ Challenges with clinical diagnosis and limitations with imaging in obesity may have led to the underdiagnosis of DVT and PE in our participants. Resolution of DVTs and underestimation of outcomes could have occurred because of routine thromboprophylaxis.

Several studies have investigated sex-specific differences in the occurrence of VTE.^4,31,32^ Zhang et al,^4^ in their systematic review identified the female sex as a risk factor for VTE following both THA and TKA. Hypercoagulability in the immediate posttrauma phase has been reported to be more prevalent in women than men.^33^ In our study, baseline hypercoagulability was significantly more prevalent in females, and a greater number of females developed thromboembolic complications in the early phase (before hospital discharge) compared to males. Females in their middle age have been reported to carry around 220-fold increased risk of VTE in the first 6 weeks after THA/TKA when compared to middle-aged females who did not undergo surgery.^32^

Our analysis identified some interesting perioperative coagulation changes. Consistent with Hughes et al,^34^ a significant reduction in platelet count occurred. This was accompanied by an increase in fibrinogen level by POD 3 and changes in CRP. The postoperative decline in platelets may relate to the increased platelet consumption following hemostasis and due to hemodilution.^35^ As clot strength is contributed by platelets, fibrin, fibrinolysis, and factor XIII activity,^36^ it is likely that the increase in fibrinogen levels and concurrent changes in FibTEM parameters by POD 3 may be compensatory to platelet reduction.^36,37^ Elevated fibrinogen and CRP levels are also reported to occur as acute phase responses to joint arthroplasty.^38^ We also observed an increase in clot strength, increased fibrinogen levels, and increased CRP levels with increasing levels of obesity consistent with our previous study findings.^15^

To our knowledge, this is the first study exploring the predictive utility of ROTEM for VTE risk in arthroplasty patients with obesity. The strengths of our study are its prospective design and 90-day follow-up. Detailed anthropometric measurements were collected on the morning of the procedure rather than relying on past measurements. Participants were followed up until 90 days and their adherence to VTE prophylaxis was confirmed by telephone contact and by verifying the details with their general practitioners and electronic health records. The limitations of our single-center study are mainly its observational nature and a lower number of events than predicted, and this precluded any regression analyses. We used the hypercoagulability definitions previously quoted in literature^15,22^ for our primary outcome. Our study did not include validating the ROTEM-hypercoagulability by other laboratory measures,^39^ however the utility of viscoelastic assays as a sole tool in demonstrating hypercoagulability is being increasingly established.^24,40^ Our chemoprophylaxis was not standardized, and most of our patients were either on aspirin or enoxaparin. However, as per the recent Australian landmark trial, aspirin and enoxaparin prophylaxis only differed in the incidence of below-knee DVT (which is considered less important clinically), and not with PE or above-knee DVT rates.^29^ Further studies using a more restricted sample of high-risk patients, assays of thrombotic biomarkers with age-appropriate reference values, and routine postoperative DVT screening may provide more conclusive evidence for the utility of viscoelastic tests in predicting the risk of VTE.

## Conclusions

In our cohort of obese arthroplasty patients, clinically significant TE complications occurred in 1.7% of the participants before discharge and 3.3% by 90 days. Our analysis found no evidence of their associations with ROTEM-identified hypercoagulability prior to surgery. However, this was limited by the low event rate. Significant differences in inflammatory markers and perioperative markers of hypercoagulability were observed across obesity categories. Future studies are recommended to evaluate the impact of these perioperative coagulation changes on thromboembolic risk.

## Supplemental Material

sj-docx-1-cat-10.1177_10760296231199737 - Supplemental material for Association Between Thromboelastometry Identified Hypercoagulability and Thromboembolic Complications After Arthroplasty: A Prospective Observational Study in Patients With Obesity Click here for additional data file. Supplemental material, sj-docx-1-cat-10.1177_10760296231199737 for Association Between Thromboelastometry Identified Hypercoagulability and Thromboembolic Complications After Arthroplasty: A Prospective Observational Study in Patients With Obesity by Usha Gurunathan, Lily Chiang and Joel Hines, Bronwyn Pearse, Scott McKenzie, Karen Hay, Daniel Mullany, Harshal Nandurkar, Victoria Eley in Clinical and Applied Thrombosis/Hemostasis

sj-docx-2-cat-10.1177_10760296231199737 - Supplemental material for Association Between Thromboelastometry Identified Hypercoagulability and Thromboembolic Complications After Arthroplasty: A Prospective Observational Study in Patients With Obesity Click here for additional data file. Supplemental material, sj-docx-2-cat-10.1177_10760296231199737 for Association Between Thromboelastometry Identified Hypercoagulability and Thromboembolic Complications After Arthroplasty: A Prospective Observational Study in Patients With Obesity by Usha Gurunathan, Lily Chiang and Joel Hines, Bronwyn Pearse, Scott McKenzie, Karen Hay, Daniel Mullany, Harshal Nandurkar, Victoria Eley in Clinical and Applied Thrombosis/Hemostasis
