# Supplementary material for: Association Between Thromboelastometry Identified Hypercoagulability and Thromboembolic Complications After Arthroplasty: A Prospective Observational Study in Patients With Obesity
Source: Clin Appl Thromb Hemost. 2023 Oct 9;29:10760296231199737. doi: 10.1177/10760296231199737 (PMC10566273; doi:10.1177/10760296231199737)
Supplement: sj-docx-1-cat-10.1177_10760296231199737 - Supplemental material for Association Between Thromboelastometry Identified Hypercoagulability and Thromboembolic Complications After Arthroplasty: A Prospective Observational Study in Patients With Obesity [file sj-docx-1-cat-10.1177_10760296231199737.docx]

**Supplementary table 1:** **Coagulation parameters in participants based on their thromboembolic complication status**

| **Coagulation parameters** | **In-hospital thromboembolic complications (n=303)** | | |
| --- | --- | --- | --- |
|  | **No (n=298)** | **Yes (n=5)** | ***P-*value** |
| **Baseline values** | | | |
| Fibrinogen, g/l^a^ | 3.4 (2.9-3.9) | 3.2 (2.6-3.7) | 0.46 |
| Platelets, x 10^9^ /l^b^ | 253.6 (61.3) | 215.0 (68.5) | 0.16 |
| ExTEM CT (s)^a^ | 64.0 (60.0-70.0) | 57.0 (57.0-61.0) | 0.16 |
| ExTEM CFT (s)^a^ | 69.0 (59.0-85.0) | 80.0 (66.0-86.0) | 0.62 |
| ExTEM MCF (mm) ^a^ | 67.0 (63.0-69.0) | 61.0 (60.0-65.0) | 0.16 |
| ExTEM MCF G score (K dyne/cm^2^) ^b^ | 9.84 (2.1) | 8.45 (2.5) | 0.15 |
| ExTEM A10 (mm) ^a^ | 57.0 (53.0-61.0) | 54.0 (51.0-54.0) | 0.30 |
| InTEM CT (s) ^a^ | 174.0 (161.0-186.0) | 180.0 (167.0-181.0) | 0.81 |
| InTEM CFT (s) ^a^ | 72.0 (59.0-90.0) | 76.0 (67.0-82.0) | 0.82 |
| InTEM MCF (mm) ^a^ | 63.0 (59.0-66.0) | 59.0 (59.0-62.0) | 0.27 |
| InTEM A10 (mm) ^a^ | 55.0 (50.0-58.0) | 52.0 (51.0-53.0) | 0.43 |
| FibTEM CT (s) ^a^ | 64.0 (60.0-68.0) | 60.0 (51.0-62.0) | 0.083 |
| FibTEM CFT (s)^1 a^ | 728.0 (332.0-1597.0) | 965.0 (633.0-1297.0) | 0.73 |
| FibTEM MCF (mm) ^a^ | 16.0 (14.0-19.0) | 18.0 (16.0-20.0) | 0.54 |
| FibTEM A10 (mm) ^a^ | 15.0 (13.0-18.0) | 17.0 (15.0-19.0) | 0.40 |
| **Postoperative values*** | | | |
| Fibrinogen, g/l ^a^ | 7.5 (6.5-8.6) | 7.6 (7.4-7.6) | 0.91 |
| Platelets, x 10^9^ l^-1 b^ | 211.0 (51.6) | 151.4 (37.8) | 0.011 |
| ExTEM CT (s) ^a^ | 64.5 (59.0-71.0) | 59.0 (58.0-67.0) | 0.66 |
| ExTEM CFT (s) ^a^ | 47.0 (41.0-55.0) | 45.0 (43.0-51.0) | 0.91 |
| ExTEM MCF (mm) ^a^ | 72.0 (69.0-74.0) | 70.0 (69.0-73.0) | 0.63 |
| ExTEM A10 (mm) ^a^ | 65.0 (62.0-68.0) | 64.0 (63.0-67.0) | 0.77 |
| ExTEM MCF G score (K dyne/cm^2^)^b^ | 1.27 (2.5) | 1.21 (3.4) | 0.59 |
| InTEM CT (s) ^a^ | 162.0 (152.0-170.0) | 175.0 (163.0-178.0) | 0.094 |
| InTEM CFT (s) ^a^ | 48.5 (43.0-57.0) | 48.0 (37.0-49.0) | 0.20 |
| InTEM MCF (s) ^a^ | 69.0 (66.0-71.0) | 67.0 (64.0-70.0) | 0.56 |
| InTEM A10 (mm) ^a^ | 62.0 (58.0-65.0) | 61.0 (57.0-63.0) | 0.64 |
| FibTEM CT (s) ^a^ | 64.0 (58.0-71.0) | 63.0 (58.0-65.0) | 0.57 |
| FibTEM CFT (s) ^a^ | 91.0 (61.5-145.5) | 71.0 (56.0-143.0) | 0.57 |
| FibTEM MCF (mm) ^a^ | 29.0 (27.0-33.0) | 32.0 (27.0-35.0) | 0.40 |
| FibTEM A10 (mm) ^a^ | 26.0 (24.0-29.0) | 28.0 (24.0-31.0) | 0.48 |
| **Temporal changes from pre to postoperative (percentage changes)** | | | |
| Fibrinogen, g/l ^a^ | 114.8 (76.7-155.2) | 103.8 (95.6-157.6) | 0.80 |
| Platelets, x 10^9^ /l ^b^ | -16.1 (11.6) | -28.0 (72) | 0.022 |
| ExTEM CT (s) ^a^ | -1.6 (-9.8-12.7) | 3.5 (1.8-18.4) | 0.38 |
| ExTEM CFT (s) ^a^ | -29.8 (-42.7--20.0) | -40.7 (-43.8--29.5) | 0.59 |
| ExTEM MCF (mm) ^a^ | 7.8 (4.3-11.6) | 15.4 (13.1-16.7) | 0.076 |
| ExTEM A10 (mm) ^a^ | 13.6 (8.1-19.6) | 22.7 (16.7-25.5) | 0.20 |
| ExTEM MCF G score (K dyne/cm^2^) ^b^ | 33.2 (4.1) | 45.4 (2.6) | 0.51 |
| InTEM CT (s) ^a^ | -6.8 (-12.8-2.6) | -9.0 (-9.9-6.6) | 0.67 |
| InTEM CFT (s) ^a^ | -30.4 (-40.6--20.3) | -39.0 (-44.8--35.5) | 0.25 |
| InTEM MCF (mm) ^a^ | 8.2 (4.6-13.2) | 13.6 (8.5-22.6) | 0.19 |
| InTEM A10 (mm) ^a^ | 13.1 (6.9-20.0) | 19.6 (9.6-30.0) | 0.39 |
| FibTEM CT (s) ^a^ | 0.0 (-8.5-10.9) | 7.4 (4.8-7.8) | 0.22 |
| FibTEM CFT (s) ^a^ | -86.5 (-95.8--76.5) | -93.8 (-96.5--91.2) | 0.35 |
| FibTEM MCF (mm) ^a^ | 76.2 (50.0-100.0) | 77.8 (62.5-80.0) | 0.82 |
| FibTEM A10 (mm) ^a^ | 68.8 (46.7-92.9) | 64.7 (55.0-68.4) | 0.84 |

*Values are in ^a^median (IQR) with P- values from Wilcoxon’s rank-sum test or ^b^mean (SD) with P- values from unpaired t-test.*

**Missing data >15% for all postoperative values except fibrinogen levels*

*Reference ranges: ExTEM: CT: 50-80 s; CFT: 46-149 s; A10: 43-63 mm; MCF: 55-72 mm; InTEM: CT: 161-204 mm; CFT: 62-130 s; A10: 43-62 mm; MCF: 51-69 mm; FibTEM: CT: 46-84s; CFT: not reported; A10: 6-21 mm; MCF: 6-21 mm. CT: Clotting time; CFT: Clot formation time; A10: Amplitude at 10 minutes; MCF: Maximum clot firmness.*
