# Supplementary material for: Association Between Thromboelastometry Identified Hypercoagulability and Thromboembolic Complications After Arthroplasty: A Prospective Observational Study in Patients With Obesity
Source: Clin Appl Thromb Hemost. 2023 Oct 9;29:10760296231199737. doi: 10.1177/10760296231199737 (PMC10566273; doi:10.1177/10760296231199737)
Supplement: sj-docx-2-cat-10.1177_10760296231199737 - Supplemental material for Association Between Thromboelastometry Identified Hypercoagulability and Thromboembolic Complications After Arthroplasty: A Prospective Observational Study in Patients With Obesity [file sj-docx-2-cat-10.1177_10760296231199737.docx]

**Supplementary table 2: Characteristics of the participant across BMI categories. Values are in ^a^mean (SD) with *P* -values from unpaired t-test, ^b^median (IQR) with *P* -values from Wilcoxon’s rank-sum test, or ^c^ n (%) with *P* -values from Pearson’s chi-square test or ^d^Fisher’s exact test**

| **Parameters** | **BMI (**kg/m^2^**)** | | | |  |
| --- | --- | --- | --- | --- | --- |
|  | <30 kg/m^2^ and/or high waist circumference*  (n=71) | 30 – 34.99 kg/m^2^ (n=104) | 35-39.99 kg/m^2^  (n=93) | >40 kg/m^2^  (n=35) | *P*- value |
| Age (years)^a^ | 68.6 (9.6) | 67.1 (9.9) | 66.0 (9.0) | 62.8 (8.7) | 0.024 |
| Female^c^ | 26 (36.6) | 57 (54.8) | 61 (65.6) | 27 (77.1) | <0.001 |
| Waist circumference (cm)^a^ | 98.5 (8.6) | 105.0 (9.1) | 112 (9.7) | 122 (12.7) | <0.001 |
| Hip Circumference(cm)^b^ | 105 (101.0-108.0) | 110.0 (106.0-115.0) | 121.0 (115.5-127.0) | 133.0 (123.0-142.0) | <0.001 |
| Neck circumference (cm)^a^ | 38.4 (3.5) | 39.1 (4.1) | 39.6 (3.9) | 40.6 (3.8) | 0.05 |
| WHR^a^ | 0.9 (0.1) | 1.0 (0.1) | 0.9 (0.1) | 0.9 (0.1) | 0.34 |
| Surgery^c^ |  |  |  |  | <0.001 |
| THA | 36 (50.7) | 30 (28.8) | 28 (30.1) | 5 (14.3) |  |
| TKA | 35 (49.3) | 74 (71.2) | 65 (69.9) | 30 (85.7) |  |
| Type of surgery ^c^ |  |  |  |  | 0.004 |
| Non-robotic | 45 (63.4) | 59 (56.7) | 71 (76.3) | 29 (82.9) |  |
| Robotic | 26 (36.6) | 45 (43.3) | 22 (23.7) | 6 (17.1) |  |
| ASA status > 2^c^ | 19 (26.8) | 42 (40.4) | 41 (44.1) | 29 (82.9) | <0.001 |
| Comorbidities |  |  |  |  |  |
| Active cancer^c^ | 5 (7.0) | 6 (5.8) | 9 (9.7) | 3 (8.6) | 0.75 |
| Current smoking^d^ | 4 (5.6) | 2 (1.9) | 5 (5.4) | 1 (2.9) | 0.50 |
| CCF^d^ | 0 (0.0) | 4 (3.8) | 2 (2.2) | 1 (2.9) | 0.36 |
| IHD^c^ | 7 (9.9) | 8 (7.7) | 4 (4.3) | 2 (5.7) | 0.54 |
| COPD^c^ | 4 (5.6) | 7 (6.7) | 4 (4.3) | 3 (8.6) | 0.73 |
| OSA^c^ | 9 (12.7) | 20 (19.2) | 23 (24.7) | 14 (40.0) | 0.013 |
| CVA^d^ | 1 (1.4) | 6 (5.8) | 3 (3.2) | 2 (5.7) | 0.42 |
| CKD^d^ | 7 (10.0) | 11 (11.0) | 7 (7.8) | 4 (11.4) | 0.87 |
| HTN^c^ | 34 (47.9) | 63 (60.6) | 56 (60.2) | 22 (62.9) | 0.30 |
| DM^c^ | 7 (9.9) | 23 (22.1) | 20 (21.5) | 11 (31.4) | 0.041 |
| Baseline CRP (mg/l) ^b^ | 1.5 (1.5-3.8) | 2.9 (1.5-6.1) | 3.6 (1.5-7.8) | 5.1 (2.8-7.9) | 0.002 |
| Metabolic syndrome^c, 2^ | 21 (29.6) | 54 (51.9) | 47 (50.5) | 21 (60.0) | 0.006 |
| Baseline lipid profile^b^ |  |  |  |  |  |
| HDL (mmol/l) | 1.3 (1.1-1.6) | 1.3 (1.1-1.5) | 1.2 (1.0-1.5) | 1.2 (1.1-1.4) | 0.077 |
| LDL (mmol/l) | 2.7 (1.6-3.5) | 2.7 (2.0-3.5) | 3.0 (2.0-3.9) | 2.2 (1.6-3.0) | 0.024 |
| Triglycerides (mmol/l) | 1.4 (0.9-2.0) | 1.6 (1.1-2.4) | 1.6 (1.1-2.3) | 1.8 (1.2-2.4) | 0.26 |
| Cholesterol (mmol/l) | 4.8 (3.7-5.8) | 4.7 (4.0-5.6) | 4.9 (4.1-6.0) | 4.3 (3.7-5.0) | 0.049 |
| D dimer (μg/ml) (n=256) ^b^ | 1.6 (1.3-2.1) | 1.7 (1.3-2.2) | 1.5 (1.3-2.2) | 1.6 (1.3-1.8) | 0.62 |
| Elevated hs-cTnI ^‡^, ^c^ (n=125) | 6 (15.4) | 6 (16.2) | 8 (22.2) | 1 (7.7) | 0.73 |
| Duration of surgery (min)^b^ | 117.0 (98.0-130.0) | 113 (94.0-132) | 103.0 (90.0-122.0) | 105.0 (95.0-130.0) | 0.16 |
| Postoperative length of stay (days)^b^ | 3.0 (3.0-4.0) | 4.0 (3.0-4.0) | 3.0 (3.0-4.0) | 4.0 (3.0-5.0) | 0.021 |

**There were three participants with BMI 24-25 kg/m^2^ but with high waist circumference, defined as >80 cm(female); >94 cm (male)*

*† International consensus definition [5]*

*‡ Elevated hs-cTnI: ≥ 10 ng/l (female) and ≥ 20 ng/l (male) with an increase from baseline levels of at least 5 units*

*THA: total hip arthroplasty; TKA: total knee arthroplasty; ASA: American Society of Anesthesiologists; CCF: Congestive cardiac failure; IHD: Ischaemic heart disease; COPD: Chronic obstructive pulmonary disease; OSA: Obstructive sleep apnoea; CKD: Chronic kidney disease; HTN: Hypertension; DM: Diabetes mellitus; CRP: C-reactive protein; IDF: International Diabetes Federation; HDL: High density lipoprotein; LDL: low density lipoprotein; hs-cTnI: High sensitivity cardiac troponin.*
